# Demonstration of IS*711 *transposition in *Brucella ovis *and *Brucella pinnipedialis*

**DOI:** 10.1186/1471-2180-8-17

**Published:** 2008-01-24

**Authors:** Alain A Ocampo-Sosa, Juan M García-Lobo

**Affiliations:** 1 Departamento de Biología Molecular, Universidad de Cantabria, Instituto de Biomedicina y Biotecnología de Cantabria, IBBTEC. (CSIC – Universidad de Cantabria – IDICAN), Santander. Spain

## Abstract

**Background:**

The *Brucella *genome contains an insertion sequence (IS) element called IS*711 *or IS*6501*, which is specific to the genus. The copy number of IS*711 *varies in the genome of the different *Brucella *species, ranging from 7 in *B. abortus*, *B. melitensis *and *B. suis *to more than 30 in *B. ovis *and in *Brucella *strains isolated from marine mammals. At present, there is no experimental evidence of transposition of IS*711*, but the occurrence of this element with a high copy number in some species, and the isolation of *Brucella *strains with "ectopic" copies of IS*711 *suggested that this IS could still transpose.

**Results:**

In this study we obtained evidence of transposition of IS*711 *from the *B. ovis *and *B. pinnipedialis *chromosomes by using the "transposon trap" plasmid pGBG1. This plasmid expresses resistance to tetracycline only if the repressor gene that it contains is inactivated. The strains *B. melitensis *16 M, *B. abortus *RB51, *B. ovis *BOC22 (field strain) and *B. pinnipedialis *B2/94, all containing the plasmid pGBG1, were grown in culture media with tetracycline until the appearance of tetracycline resistant mutants (Tc^R^). Tc^R ^mutants due to IS*711 *transposition were only detected in *B. ovis *and *B. pinnipedialis *strains.

**Conclusion:**

Four different copies of IS*711 *were found to transpose to the same target sequence in the plasmid pGBG1. This demonstrated that IS*711 *are active *in vivo*, specially in *Brucella *species with a high number of IS*711 *copies as *B. ovis *and *B. pinnipedialis*.

## Background

Bacteria in the genus *Brucella *are the etiological agents of brucellosis, one of the most important zoonotic diseases, affecting human and domestic animals of high economic and social value. The genus contains several species that are defined mainly on the basis of animal host specificity [1]. A growing interest in development of tools for molecular epidemiology and control of brucellosis has resulted in a large amount of published genome sequence data. The complete genome sequences of *B. melitensis *[2], *B. suis *[3], and two *B. abortus *strains [4,5] are already published, and the genome sequences of *B. ovis*, *B. canis *and the *B. abortus *vaccine strain S19 are also completed but not published yet.

The genus *Brucella *bears an Insertion sequence (IS) element, originally detected in *Brucella ovis *as a repeated polymorphic DNA sequence in 1990 [6]. Subsequently, this repeat was proposed to be an IS based on its genetic organisation, which is similar to that of some elements described before, and named as IS*711 *[7] or IS*6501 *[8]. IS elements are defined as small segments of DNA with a simple genetic organisation and capable of inserting at multiple sites into target DNA molecules. The best known ISs are those of the Gram-negative bacteria, but these mobile elements have been widely reported in a variety of bacterial genera. A compilation of the available data on insertion sequences can be found in the IS database [9].

IS*711 *is an 842 bp element bounded by 20 bp imperfect inverted repeats. This IS contains two overlapping open reading frames, which are believed to translate into a single polypeptide by means of a -1 frameshift of the translation apparatus, a feature also present in other ISs [10,11]. IS*711 *appears to duplicate the dinucleotide TA of the consensus target site, YTAR [7].

IS*711 *is exclusively present in the genome of all described species of the genus *Brucella*. The sequenced genomes of closely related α-proteobacteria do not contain an IS obviously related to IS*711*, suggesting that this insertion sequence is an element native to the genus *Brucella*. Furthermore IS*711 *has been shown to be associated with the repeated elements BRU-RS1 and BRU-RS2 that could represent insertion hot spots for this IS [12,13].

Some published work already suggested that IS*711 *could be an active transposon [14]. The copy number of IS*711 *is different in the species of the genus. *B. melitensis *has 7 copies of this element; the same number as *B. abortus *and *B. suis*. However, *B. ovis *contains about 35 copies [7,8], and the recently discovered *Brucella *from marine mammals carry more than 20 IS*711 *copies [15]. Furthermore, the finding of a new IS*711 *copy inactivating the *wboA *gene in the vaccine strain RB51[14] also indicated that IS*711 *could be an active element. However, there is no experimental evidence of IS*711 *transposition up to now. Due to its proven genomic stability, IS*711 *has been used as a target to identify *Brucella *species and biovars [12,16].

Other ISs have been described in *Brucella *in addition to IS*711*, such as the elements named IS*Bm1*, IS*Bm2 *and IS*Bm3 *[17] and IS*Bm4 *[18], as well as IS*2020*, which forms part of the composite transposon Tn*2020 *[19], but the mobility of these elements has not been observed experimentally.

In this study we report evidence for IS*711 *transposition from *B. ovis *and *B. pinnipedialis *by using pGBG1, a plasmid for isolating mobile DNA elements [20]. This constitutes the first experimental report of activity of a transposon in the genus *Brucella*.

## Results and discussion

### Isolation and characterisation of tetracycline resistant derivatives of PGBG1 in *Brucella*

The transposon trap plasmid pGBG1 was introduced into *Brucella *by conjugation as described in the Materials and Methods. Plasmid PGBG1 carries a tetracycline resistance gene whose transcription is blocked by the repressor of bacteriophage lambda. Insertions of resident transposons into the repressor gene result in transcription of the Tc^R ^gene of pGBG1 and can be selected on tetracycline containing media. As a preliminary step for the transposition experiments, we determined the stability of pGBG1 and the MIC of tetracycline for the different *Brucella *species to be used, since this plasmid had not been employed before in this genus. As result of these studies we found that *Brucella *strains must be grown in the presence of chloramphenicol in order to maintain pGBG1, and also determined that the most appropriate tetracycline concentration to avoid the appearance of spontaneous mutants in plasmid free *Brucella *was 30 μg ml^-1^. To assay for transposition, various independent colonies (10–20) of each *Brucella *strain carrying pGBG1 were grown in appropriate media. Cultures (0.1 ml) were then plated separately on agar plates with 30 μg ml^-1 ^of tetracycline or with 25 μg ml^-1 ^of chloramphenicol (after dilution), and incubated at 37°C in 5% CO_2 _atmosphere until growth was observed.

**Figure 1:**
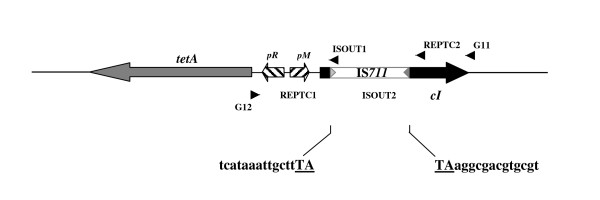
**Schematic drawing showing the selection cartridge of the plasmid pGBG1 after the insertion of IS*711***. *Brucella *strains containing these plasmids were resistant to tetracycline. The IS*711 *insertion is represented by a white box flanked by grey arrowheads. The target sequence is indicated and the duplicated bases appear as underlined capital letters. The primers used to amplify and determine the precise location of insertions in the selection cartridge are indicated with the primer name and black arrow heads.

**Figure 2:**
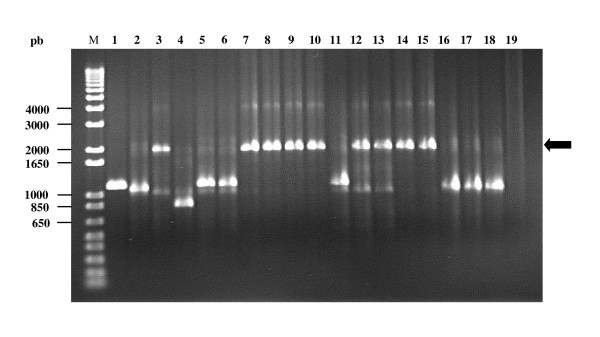
**PCR analysis of the plasmids found in tetracycline resistant mutants**. PCR with primers G11 and G12 was carried out in order to amplify the selection cartridge of pGBG1 in tetracycline resistant mutants obtained in *Brucella*. The black arrow indicates the position of the PCR fragment obtained from pGBG1/Tc^R ^mutants due to insertions encountered in *B. ovis *BOC22 and *B. pinnipedialis *B2/94 strains (Lanes 3, 7, 8, 9, 10, 12, 13, 14 and 15). Mutants due to deletions and point mutations (Lanes 2, 4, 5, 6, 11, 16, 17 and 18) produced PCR products of the same size or smaller than the vector pGBG1 (Lane 1). M: Molecular Weight Marker 1 Kb plus DNA Ladder.1. Lane 19. Negative control (PCR reaction mixture without DNA).

The frequencies of occurrence of tetracycline resistant colonies relative to chloramphenicol resistant CFU's ranged from 10^-7 ^to 10^-9^, depending on the strain (Table 1). To determine whether the appearance of tetracycline resistant colonies was due either to transposition into pGBG1 of a *Brucella *transposon or to any other mutational event, selected colonies from each independent experiment were analysed by PCR using the primers G11 and G12 (Fig. 1, Table 2), which amplified the selection cartridge of pGBG1 plasmid as a 1.2 kb fragment. Some of the tetracycline resistant isolates produced a PCR amplification fragment of around 2.0 kb. The size of these DNA fragments represented an increase of approximately 0.8 kb relative to the size of the target region of pGBG1, which was consistent with the integration of a copy of IS*711 *into the repressor controlling the tetracycline resistance gene of pGBG1. From the four *Brucella *strains in which the transposon trap pGBG1 was used, only *B. ovis *BOC22 and *B. pinnipedialis *B2/94 produced tetracycline resistant plasmids containing insertions (Fig. 2). No tetracycline resistant colonies containing insertions in the experiments that used *B. abortus *or *B. melitensis *as hosts were obtained. Some tetracycline resistant colonies were obtained with these two donors. PCR analysis of the corresponding plasmids in these cases, (and in some colonies from *B. ovis *and *B. pinnipedialis *as well) could not be explained by integration events. These resistant mutants probably resulted from point mutations (when the size of target region was apparently unchanged) or from deletions (when the size of target region decreased) in the pGBG1 selection cartridge (Fig. 1).

**Table 1:** Frequencies of appearance of Tc^R ^mutants in *Brucella *containing pGBG1

Strain	Frequency of Tc^R ^mutants*	Number of Tc^R ^mutants analysed	Number of mutants with insertions	% of insertions detected	Mobile element involved
*B. ovis *BOC22	(20) 8.6 × 10^-8^	52	30	57.7	IS*711*
*B. abortus *RB51	(10) 2.9 × 10^-9^	46	0	0	none
*B. melitensis *16M-N	(10) 3.05 × 10^-7^	13	0	0	none
*B. pinnipedialis *B2/94	(10) 5.0 × 10^-8^	35	4	11.4	IS*711*

* Average frequency of all experiments. The number of independent experiments is in parentheses

**Table 2:** Oligonucleotide primers

Primer name	Sequence 5'-3'	Reference
711u	CACAAGACTGCGTTGCCGACAGA	This study
711d	CATATGATGGGACCAAACACCTAGGG	This study
G11	TATCAGCTATGCGCCGACCAGAAC	[20]
G12	GCCAATCCCCATGGCATCGAGTAA	[20]
REPTC1	GGCGTTATAAGCATTTAATG	This study
REPTC2	AAAAAGAAACCATTAACACA	This study
ISOUT1	GCGGCCGGGCGTACCAACTCG	This study
ISOUT2	GCTATCGTCGTATTGCGCTGC	This study

### Identification of IS*711 *in pGBG1 Tc^R ^insertions

**Figure 3:**
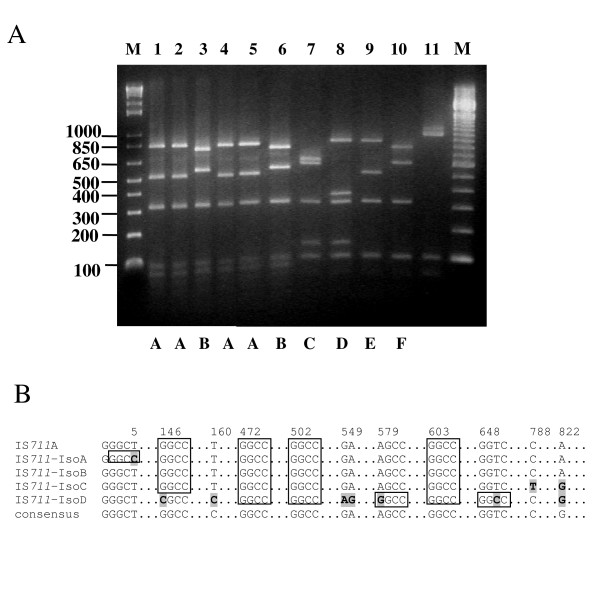
**Diversity of restriction profiles and nucleotide sequence of IS*711 *copies recovered in IS*711 *insertions in pGBG1**. A) PCR products from several pGBG1/Tc^R ^mutants digested with *Hae*III. Lanes 1 to 6 contain insertions obtained in *B. pinnipedialis *B2/94. Lanes 7–10 insertions obtained in *B. ovis *BOC22 Lane 11, PCR product from pGBG1. M: Molecular size marker (1 Kb DNA ladder, Invitrogen). Letters under the picture indicate the different restriction patterns obtained. In B) the nucleotide sequences of the IS*711 *copies transposed into *cI *lambda repressor gene were compared with IS*711*A from *B. ovis *(GenBank Accession number: M94960). Only the variable regions and the *Hae*III sites are shown. Identical nucleotides are represented by dots. Numerals in the top indicate the positions where nucleotide changes occur. Nucleotide substitutions are boldface and shaded. The *Hae*III restriction sites are indicated within open boxes.

As a first step towards identification of the DNA inserted into the tetracycline resistant insertional mutants, the PCR products were digested with the restriction enzyme *Hae*III. Six different restriction patterns were observed, four in *B. ovis *BOC22 (C, D, E and F) and two in *B. pinnipedialis *B2/94 (A and B, Fig. 3A).

**Figure 4:**
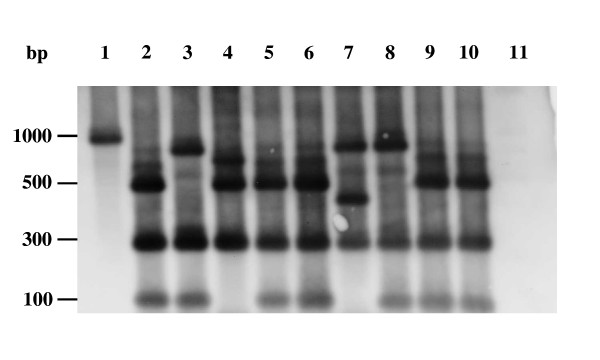
**Southern blot hybridisation of amplified regions from Tc^R ^mutants showing the insertion of IS*711***. PCR products obtained with primers G11 and G12 from several pGBG1/Tc^R ^insertion mutants were digested with *Hae*III and hybridised with an IS*711 *probe. Lane 1: IS*711 *probe, lanes 2–10: insertion mutants containing IS*711*, lane 11: PCR product from pGBG1. Molecular sizes of the fragments are indicated in bp.

To confirm whether the insertions were due to integration of IS*711 *into pGBG1, the *Hae*III digested PCR products containing insertions were transferred to a nylon membrane and hybridised with an IS*711 *probe. Southern hybridisation analysis showed that all insertions in pGBG1 were due to the presence of IS*711 *(Fig. 4).

The different restriction patterns obtained with *Hae*III could indicate that the IS had inserted in different positions of the target gene, but could also be the result of IS*711 *sequence polymorphism in *B. ovis *and *B. pinnipedialis*, as both species are poorly characterised. In order to clarify this point, the PCR products obtained from selected tetracycline resistant insertional mutants (pGBG1::IS*711*) were purified and sequenced with the ISOUT1/ISOUT2, and the REPTC1/REPTC2 pairs of primers (Fig. 1 and Table 2).

### Sequence analysis of the IS*711 *inserted copies

Twenty one PCR products from pGBG1::IS*711 *tetracycline resistant mutants were selected to determine the complete sequence of the IS*711 *copy which integrated into *cI *gene. The sequence analysis showed that all the IS*711 *insertions had taken place at the same point within the *cI *gene, near its 5' terminal end. In all cases the insertion occurred at the sequence TTAA according to the predicted IS*711 *target. The central dinucleotide TA was duplicated at both sides of the element as was expected. IS711 insertions at this point occurred in the two possible orientations. It was surprising to find that all the IS*711 *insertions occurred in the same point of the *cI *gene. Transposons vary in the degree of target specificity from highly specific (i.e. Tn*7*) to near random insertion transposons [21]. The observance of a single insertion site among 40 independent transposition experiments indicated a strong degree of specificity in target selection. The reported target for IS*711 *integration was the very frequent YTAR sequence. However, a preference for insertion of IS*711 *into the Bru-RS palindromic repeats has also been reported, indicating the existence of some undetermined determinants of integration specificity. Our experimental system can only detect insertions into the 1 kb *cI *gene, which contains around fifteen YTAR sites. The reason for the observed transposition preference for a single site remains unexplained.

Sequence analysis indicated the presence of four different copies of IS*711 *disrupting the *cI *gene. Each copy was designated with a different nomenclature: IS*711*-IsoA, IS*711*-IsoB, IS*711*-IsoC and IS*711*-IsoD. Nucleotide sequences of these IS*711 *copies were compared with the copy named IS*711*A from *B. ovis *(GenBank Accession number: M94960). The copy designated as IS*711*-IsoB was identical to IS*711*A. Differences between other IS*711 *copies were due to a few nucleotide substitutions that in some cases led to the appearance or disappearance of some of *Hae*III restriction sites (Figure 3b). These sequence differences were responsible of the remaining diversity in *Hae*III digestions shown in Fig. 3. Some of these nucleotide changes were silent and the putative transposases of the different IS*711 *copies were identical to that of IS*711*A, with the exception of IS*711*-IsoD which was different to other copies by 3 amino acids which were Gly in place of Arg, Lys replacing Glu and Val instead of Ala.

These results demonstrate that some of the IS*711 *copies in *B. ovis *and *B. pinnipedialis *were transpositionally active even when the observed transposition frequency was very low. We were unable to precisely determine transposition frequencies with this assay but if we consider that the tetracycline resistant colonies selected for analysis were representative of the whole population, we know that 57.7% of the mutants obtained in *B. ovis *and 11.4% of those obtained in *B. pinnipedialis *were due to IS*711 *transposition (Table 1). These percentages allowed us to estimate the average transposition frequency around 10^-8 ^in both species. These frequencies are low when compared with other transposons.

### Transposition activity of IS*711*

Transposition of IS*711 *was detected only in *B. ovis *and *B. pinnipedialis*. We were unable to detect transposition either in *B. abortus *or in *B. melitensis *under the same experimental conditions. Many attempts to detect IS*711 *transposition in *E. coli *from plasmids containing cloned copies of IS*711 *were also unsuccessful, even when powerful detection systems as the *sacB *transposon trap [22] were used (data not shown). All these data indicated that IS*711 *transposition activity was very low, restricted to *Brucella *maybe due to the requirement for specific host factors, and only detectable in species with a high number of copies of IS*711 *per genome. The presence of some IS*711 *copies in these species encoding a full length transposase without the need for a frameshift event, could explain the observed transposition capability. However, such a copy of IS*711 *has not been observed in the genomic sequence of *B. ovis *released recently to GenBank and a gene dosage effect is a more favoured hypothesis for transposition in *B. ovis *and *B. pinnipedialis*. The requirement for many copies of the IS could be due either to a very low level of expression of the transposase, requiring an increased dose effect for activity, or to the presence of point mutations leading to inactivity of some of the copies found in *B. abortus *and *B. melitensis*. There are several reasons that could explain a low level of IS*711 *transposase expression. Firstly, that the regular initiation codon for IS*711 *OrfA is GTG, a less efficient initiation codon than the canonic ATG. Secondly, that a -1 frameshift could be necessary in order to have an active IS*711 *transposase molecule. This event is usually very inefficient with less than 5% of the translation products shifted to translate into the complete transposase. Furthermore, in some insertion sequences OrfA possesses an inhibitory action on the transposition process [23].

We examined the nucleotide sequence of the IS*711 *copies in the genomes of the three sequenced *Brucella *species and analysed them to detect the reasons for their inactivity. In some of the copies either OrfA or OrfB were truncated or altered by single nucleotide insertions or deletions. Furthermore, we found that in many of these copies the start codon for OrfA was TTG instead of GTG as happens in all the copies rescued after inactivation of the *cI *repressor in pGBG1. TTG has been reported to be less efficient than GTG as a translation initiation codon [24]. These data support that the lack of activity observed in *B. abortus *and *B. melitensis *as well as the low transposition frequency observed in *B. ovis *and *B. pinnipedialis *could be due to the low expression (translation) of the IS*711 *transposase protein.

### Distribution and analysis of IS*711 *in the *Brucella *sequenced genomes

The reported sequence of IS*711 *[7], (GenBank accession M94960) was compared with the available *Brucella *genomic sequences using local BLAST searches. The results showed the presence of 7 IS*711 *copies in *B. suis *and *B melitensis*. *B abortus *9_941 also presented 7 IS*711 *copies but one of them was severely truncated and reduced to a 100 bp terminal remnant. *B. abortus *2308 contained the same IS*711 *copies as the strain 9_941 and one additional IS*711 *copy located at exactly the same position as one of the pre-existing copies. It is well known that the *B. abortus *vaccine strain RB51, which is a derivative of 2308, contains an extra copy of IS*711 *interrupting the *wboA *gene [14].

**Figure 5:**
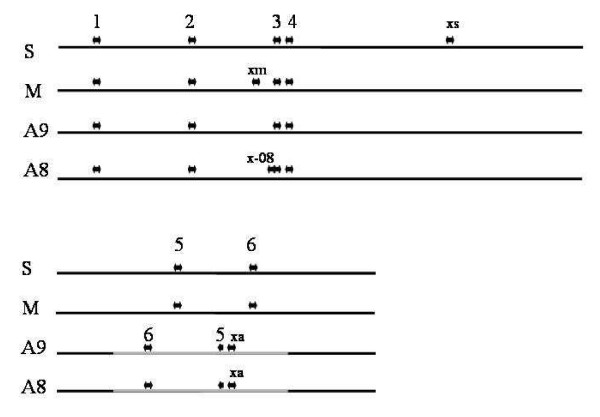
**Position of IS*711 *copies in the chromosomes of the published *Brucella *genomes**. The two blocks contain the large and the small chromosomes respectively. Key to the species: S, *B. suis*; M, *B. melitensis*; A9, *B abortus *9_941; A8, *B abortus *2308; O, *B. ovis*. Common copies numbered from 1 to 6 are marked on the top. xs, xiii, x-08 and xa are IS*711 *species specific copies. The inverted segment of *B. abortus *chromosome II is marked in grey.

The genome of *B. ovis *was known to contain a high copy number of IS*711 *estimated by hybridisation in around 30 copies [8]. The genome sequence of this species has been recently deposited in GenBank (Accesion Numbers CP000708 and CP000709). Genome sequence analysis revealed the presence of 38 complete copies of IS*711 *in the *B. ovis *genome, 25 and 13 in each chromosome respectively. Upon analysis of the genomic location of the IS*711 *copies we could determine that the position of six out of the seven copies was conserved in all the *Brucella *species. Four of the conserved copies were found in the large chromosome and two in the small chromosome. The common copies will be numbered from 1 to 6 plus a species or strain identifier (Fig. 5). When the nucleotide sequence of individual IS copies was analyzed it was evident that some sequence divergence existed among them. This indicated that IS sequence had drifted along with the other chromosomal genes in *Brucella*. At the same time this sequence variation allowed us to interpret the sequence identity between IS copies in the same organism as an indicator of recent transposition. In *B. abortus *the extra copy 711_xa was found to be identical to 711_1a. In *B. abortus *2308 there is an additional IS*711 *copy found next to 711_3a. This copy was also identical to 711_1a. These observations strongly suggest that IS711_1a could be active and also show the strong preference of IS*711 *to transpose to some sites on the genome. On the other hand the IS*711 *copy found in the *wboA *gene of the *B. abortus *vaccine strain RB51 was found to be identical to 711_a6. This finding was rather unexpected, since 711_a6 lacks the first five nucleotides from the left IR, which would usually result in loss of the transposition ability. Insertion of this copy of IS*711 *into the *wboA *gene could also be explained by a different mobility mechanism other than transposition.

If we correlate the sequence analysis with the results presented in this study, we can conclude that the natural transposition of IS*711 *occurs at very low frequency, probably due to a poor expression of IS*711 *transposase. This limitation can be overcome in *B. ovis *by the high copy number of IS*711*.

## Conclusion

In conclusion, our results demonstrate experimentally for the first time that IS*711 *is an active insertion sequence of the *Brucellae*. On the other hand, the low transpositional activity of the sequence allows the use of this element as a tool for molecular identification, at least for the species containing a low number of copies of this insertion sequence.

## Methods

### Bacterial strains plasmids and growth conditions

The bacterial strains and plasmids used in this study are listed in Table 3. *Escherichia coli *cells were cultivated overnight at 37°C in Luria-Bertani (LB) medium (Pronadisa). *Brucella *strains were grown at 37°C for 48–96 hours in a 5% CO_2 _atmosphere in *Brucella *broth (BB) (Pronadisa), Blood agar (Difco) or *Brucella *agar (BA) medium (Pronadisa). Plates were supplemented with 10% foetal bovine serum to grow *B. ovis and B. pinnipedialis*.

**Table 3:** Bacterial strains and plasmids used in this study

Strain/Plasmid	Relevant characteristics	Reference
***E. coli *strains**		
		
S17.1	Tp^R ^Sm^R^, *recA*, *thi*, *pro*, *hsd*R^-^M^+ ^RP4: 2-Tc:Mu: Km Tn7 λ *pir*	[30]
		
***Brucella *strains**		
*B. abortus *RB51	Rif^R^, rough mutant of 2308	[31]
*B. melitensis *16M-N	Spontaneous Nal^R ^mutant of ATCC 23456	This study
*B. ovis *BOC 22	Rough field strain, CO_2 _dependent	This study
*B*. pinnipedialis B2/94	*Brucella *marine strain isolated from common seal, CO_2 _dependent	[32]
		
**Plasmids**		
		
pGBG1	Cm^R^, transposon trap derivative of pBBR-MCS1	[20]

Cm^R^: Chloramphenicol resistant, Nal^R^: Nalidixic acid resistant, Rif^R^: Rifampicin resistant, Tp^R^: Trimethoprim resistant, Sm^R^: Streptomycin resistant,

pGBG1 (courtesy of M. Blot), is a derivative from the broad host-range, mobilizable plasmid pBBR1-MCS [25] containing a transposition selection module with a tetracycline resistance gene (*tetA*) whose expression is blocked by λ repressor *cI*. This plasmid allows positive selection for tetracycline resistance of transposon insertions inactivating the repressor [20].

### General DNA manipulations, PCR and Southern blot hybridisations

DNA preparations, purifications, restriction endonuclease digestions and agarose gel electrophoresis were carried out according to described standard protocols [26].

Oligonucleotide primers used in this study were purchased from MWG Biotech and are listed in Table 2. PCR reactions were usually performed in 25 μl reaction volumes containing 0.2 μM of each primer, 2.5 μl of 10× PCR reaction buffer, 1.5 mM of MgCl_2_, 0.2 mM of dNTPs (Applied Biosystems), 25 ng of template DNA and 1 unit of Bio*Taq *DNA polymerase (Bioline). Amplification was performed in a thermal cycler (GeneAmp PCR System 2400, Perkin Elmer) as follows: denaturation step at 94°C for 3 min and 25 cycles of 94°C for 30 s, 50°C for 30 s and 72°C for 1 min. After the last cycle the reaction mixture was reincubated at 72°C for 5 min.

Preparation of the IS*711 *probe was performed by PCR amplification with two primers, 711u and 711d, using genomic DNA from *B. melitensis *16 M as template. The PCR reaction was carried out as described and the amplified DNA fragment to be used as a probe was recovered from an agarose gel by using the QIAquick Gel Extraction Kit (QIAGEN). The probe was labelled with digoxigenin using a DIG-High Prime DNA Labelling and Detection Kit (Roche Diagnostics GmbH) as described by the supplier. Southern blot hybridisation with the DIG-labelled probe was performed as described before [27].

### Conjugation experiments

Introduction of plasmid pGBG1 into *Brucella *was performed by conjugation from *E. coli *S17.1. The experiments were carried out by filter mating as follows. Equal volumes (500 μl) of mid-log-phase cultures of *E. coli *S.17 (pGBG1) as donor and *B. abortus *RB51, *B. melitensis *16M-N, *B. ovis *BOC22 or *B. pinnipedialis *B2/94 as recipients were mixed and then washed with sterile PBS by centrifugation. The mixtures were spread on 0.22 μm filters (24 mm diameter, GSWP, Millipore), placed on a *Brucella *agar plate and incubated at 37°C for 12 h in 5% CO_2_atmosphere. After this period of incubation the filters with the conjugation mixtures were washed in 3 ml of BB medium. Different serial dilutions (10^-1^-10^-3^) of the conjugation mixtures were plated onto the appropriated media until growth was observed. The transconjugants of *B. melitensis *16M-N and *B. abortus *RB51 were selected on *Brucella *agar medium with chloramphenicol (25 μg ml^-1^) and either nalidixic acid (20 μg ml^-1^) or rifampicin (50 μg ml^-1^) respectively. Transconjugants of *B. ovis *BOC22 and *B. pinnipedialis *B2/94 strains were selected on BA medium supplemented with 10% of foetal bovine serum containing chloramphenicol (25 μg ml^-1^) and a mixture of antibiotics (vancomycin, 3 μg ml^-1^; colistin, 7.5 μg ml^-1^; nystatin 12.5 units ml^-1 ^and nitrofurantoin 10 μg ml^-1^) to counterselect against the donors. The presence of pGBG1 in the transconjugants was confirmed by extraction of plasmid DNA and analysis on agarose gels.

### DNA sequencing and data analysis

PCR products were purified for sequencing by using the QIAquick Gel Extraction Kit (QIAGEN). All PCR products were sequenced by our Sequencing Service using a commercial cycle sequencing kit (Perkin Elmer, Dye Terminator Cycle Sequencing Ready Fraction Kit) according to the manufacturer's conditions. Both, the nucleotide and protein sequence analysis of the different IS*711 *copies isolated from each *Brucella *strains were performed by using the Vector NTI version 5.5 computer program (Informax Inc.). Sequences were compared with the IS*711 *copies reported in GenBank with the BLAST sequence comparison tool [28].

Preliminary sequence data of the *B. ovis *genome was obtained from The Institute for Genomic Research [29]

## Authors' contributions

AOS performed most of the experimental work and wrote a draft of the paper.

JMGL designed and supervised the experimental work and wrote the paper. Both authors have read an approved the final manuscript.
